# A Simple Method for Assessment of Human Anti-Neu5Gc Antibodies Applied to Kawasaki Disease

**DOI:** 10.1371/journal.pone.0058443

**Published:** 2013-03-08

**Authors:** Vered Padler-Karavani, Adriana H. Tremoulet, Hai Yu, Xi Chen, Jane C. Burns, Ajit Varki

**Affiliations:** 1 Glycobiology Research and Training Center, Departments of Medicine and Cellular & Molecular Medicine, University of California San Diego, La Jolla, California, United States of America; 2 Department of Pediatrics, University of California San Diego, Rady Children’s Hospital, San Diego, California, United States of America; 3 Department of Chemistry, University of California Davis, Davis, California, United States of America; King’s College London, United Kingdom

## Abstract

*N*-Glycolylneuraminic acid (Neu5Gc) is an immunogenic sugar of dietary origin that metabolically incorporates into diverse native glycoconjugates in humans. Anti-Neu5Gc antibodies are detected in all human sera, though with variable levels and epitope-recognition profiles. These antibodies likely play a role in several inflammation-mediated pathologies including cardiovascular diseases and cancer. In cancer, they have dualistic and opposing roles, either stimulating or repressing disease, as a function of their dose, and some of these antibodies serve as carcinoma biomarkers. Thus, anti-Neu5Gc antibodies may signify risk of inflammation-mediated diseases, and changes in their levels could potentially be used to monitor disease progression and/or response to therapy. Currently, it is difficult to determine levels of anti-Neu5Gc antibodies in individual human samples because these antibodies recognize multiple Neu5Gc-epitopes. Here we describe a simple and specific method for detection and overall estimation of human anti-Neu5Gc antibodies. We exploit the difference between two mouse models that differ only by Neu5Gc-presence (wild-type) or Neu5Gc-absence (*Cmah^−/−^* knockout). We characterize mouse serum from both strains by HPLC, lectin and mass-spectrometry analysis and show the target Neu5Gc-epitopes. We then use *Cmah^−/−^* knockout sera to inhibit all non-Neu5Gc-reactivity followed by binding to wild-type sera to detect overall anti-Neu5Gc response in a single assay. We applied this methodology to characterize and quantify anti-Neu5Gc IgG and IgA in sera of patients with Kawasaki disease (KD) at various stages compared to controls. KD is an acute childhood febrile disease characterized by inflammation of coronary arteries that untreated may lead to coronary artery aneurysms with risk of thrombosis and myocardial infarction. This estimated response is comparable to the average of detailed anti-Neu5Gc IgG profile analyzed by a sialoglycan microarray. Both assays revealed an elevated response in acute KD patients with normal coronaries compared to patients with aneurysm or dilated coronaries. Implications of these findings are discussed.

## Introduction

Sialic acids (Sias) are 9-carbon backbone acidic sugars terminating glycan chains of various glycoproteins and glycolipids on vertebrate cell surfaces and secreted glycans. *N*-Acetylneuraminic acid (Neu5Ac) and its hydroxylated form, *N*-Glycolylneuraminic acid (Neu5Gc) are the two major Sia forms in mammals [1]–[3]. Humans are genetically Neu5Gc-deficient due to lack of the enzyme CMP-Neu5Ac hydroxylase (CMAH) [4], [5]. Similarly, a ‘human-like’ *Cmah^−/−^* knockout mouse model is Neu5Gc-deficient, in contrast to wild-type mice that contain Neu5Gc in most tissues [6]–[8]. Neu5Gc is rich in red meat and when consumed by humans it metabolically incorporates into cells as ‘self’ [9], [10], but at the same time becomes immunogenic [11]–[14]. This results in a polyclonal anti-Neu5Gc antibody response [9], [15]–[17], which we termed ‘xeno-autoantibodies’ [16]. It was also suggested that bacterial infections contribute to the generation of these anti-Neu5Gc antibodies in humans [18]. Such antibodies are proposed to contribute to xeno-transplantation rejection, in addition to anti alpha-Gal antibodies, and considered to constitute a major fraction of ‘non-Gal’ antibodies [19]. These xeno-autoantibodies can interact with tumor-associated Neu5Gc, thereby exerting dose-dependent effects: at a low dose they facilitate tumor progression via chronic inflammation [20], while at higher doses they inhibit tumor growth [21]. Furthermore, some of these antibodies are biomarkers of carcinomas and potential therapeutic agents [21]. Likewise, anti-Neu5Gc antibodies potentially play a role in vascular inflammation disease states such as atherosclerosis [22]. Thus, detection of overall anti-Neu5Gc reactivity to multiple Neu5Gc-containing epitopes is desirable as a screening method to detect risk, progression or therapeutic response in chronic inflammation-related diseases such as cancer [21].

Kawasaki disease (KD) is the leading cause of acquired pediatric heart disease in the developed world [23], [24]. It is an acute, self-llimited vasculitis associated with fever and mucocutaneous signs. At present there is no simple diagnostic test for KD and diagnosis rather relies on clinical criteria that include: prolonged high fever (>5 days), rash, conjunctival injection, cervical lymphadenopathy, changes in the oral mucosa and changes in the extremities [25], [26]. In severe cases coronary artery abnormalities (dilatation or aneurysm) are detected. KD is treated with high dose intravenous immunoglobulin G (IVIG) and aspirin, and anticoagulants for coronary artery aneurysms [25]–[28]. In KD there is a marked activation of the immune system with elevations of serum pro-inflammatory cytokines and chemokines at the acute phase [29], including elevated antibody responses to various antigens [30]–[32]. Among those, heterophile antibodies with Hanganutziu-Deicher (H-D) specificity were shown to be elevated in KD patients [33]–[35]. In those generic assays the H-D antigen was not well defined, but was suggested to contain Neu5Gc [34], [35]. Thus, we hypothesized that anti-Neu5Gc antibodies response can be detected in KD patients and their levels could perhaps be associated with disease severity and/or progression.

Currently, it is difficult to determine the overall level of anti-Neu5Gc antibodies in individual human samples because these antibodies recognize multiple Neu5Gc-epitopes that contain terminal Neu5Gc at the tips of their glycan chains. Available assays to detect human anti-Neu5Gc antibodies rely on the difference between Neu5Ac and Neu5Gc, and require matched sets of Neu5Ac-epitopes and Neu5Gc-epitopes. The final specific Neu5Gc-reactivity is determined after subtraction of the matching Neu5Ac-reactivity [9], [15], [16], [21]. This method is likely to generate some ‘false-negative’ signals because a fraction of anti-Neu5Gc antibodies may cross-react with Neu5Ac and/or because anti-Neu5Ac antibodies may be unrelated. In addition, such antibodies with low affinity may overlap by cross-reacting with several Neu5Gc-containing epitopes, as supported by ELISA inhibition assays [16]. Mild periodate treatment, which truncates the side chain of unmodified sialic-acid (9-*O*-acetylated Sia are resistant), can also be used to ascribe Sia-specific binding but it is not Neu5Gc-specific [21], [36]. Alternatively, we previously described sialoglycan microarrays with several individual Neu5Gc-epitopes for detection of only a fraction of the overall anti-Neu5Gc response for each unique Neu5Gc-epitope [21], [37]. All these methods require laborious and specific chemical synthesis of matched Neu5Ac/Neu5Gc-pairs, and even then may be limited to mono-specificities of certain Neu5Gc-epitopes. In contrast, we here describe a novel robust assay that is simple, rapid and specific to get an estimate of overall anti-Neu5Gc antibodies against multiple Neu5Gc-epitopes. We apply this assay to evaluate anti-Neu5Gc responses in Kawasaki disease patients and controls, and show it to be comparable to the more sophisticated sialoglycan microarray assay. Both assays revealed an elevated response in acute KD patients with normal coronaries compared to patients with aneurysm or dilated coronaries.

## Materials and Methods

### Ethics

Mice were maintained according to UCSD Institutional Animal Care and Use Committee guidelines for laboratory animals (IACUC permit S01227: “Evaluation of the Role of Glycans in Normal Physiology, Malignancies and Immune Responses”). Mice were euthanized by CO_2_ inhalation with subsequent cardiac bleeding. Human patients were enrolled at Rady Children’s Hospital, San Diego after obtaining written parental informed consent and subject assent, as appropriate. The study protocol “Human Research Protection Program” was reviewed and approved by the UCSD Institutional Review Board. All data were analyzed anonymously.

### Antibodies and Lectins

We used purified human IgA, purified human IgG, horseradish peroxidase (HRP)-goat-anti-mouse IgG (Fcγ fragment specific), HRP-streptavidin, Cy3-goat-anti-human IgG (H+L) (Jackson ImmunoResearch Laboratories), HRP-conjugated goat-anti-human IgA (Calbiochem), HRP-goat-anti-human IgG (Bio-Rad) and biotinylated lectins SNA, MAL-1 and MAL-2 (Vector Labs).

### Mice

Wild-type (WT) and *Cmah* ^−/−^ (*Cmah*-KO) mice [6] were bred in a congenic C57BL/6 background and maintained according to Institutional Animal Care and Use Committee guidelines for laboratory animals (IACUC permit S01227: Evaluation of the Role of Glycans in Normal Physiology, Malignancies and Immune Responses). Mouse sera were obtained by terminal cardiac bleeding.

### Mice Sera Screening

Only mice sera free of mouse-anti-human IgG antibodies were used. To screen for such sera, Costar 96-well plates were coated with human IgG at 1 µg/well in coating buffer (50 mM sodium carbonate-bicarbonate buffer, pH 9.5) then incubated overnight at 4°C. Wells were blocked for 2 hours at room temperature (RT) with TBST (150 mM NaCl, 50 mM Tris pH 8, 0.1% Tween-20). After removal of TBST, candidate mouse sera (diluted in TBST at 1∶400) were added to triplicate wells at 100 µl/well then incubated at RT for 2 hours. Wells were washed three times with TBST then detection antibody (HRP-goat anti-mouse IgG Fcγ fragment specific, diluted 1∶10,000 in TBST) was added at 100 µl/well and incubated for 1 hour at RT. After washing three times with TBST, wells were developed with *O*-phenylenediamine (0.5 mg/ml) in citrate-PO_4_ buffer, pH 5.5, reaction was stopped with H_2_SO_4_ and absorbance was measured at 490 nm wavelength on a SpectraMax M3 (Molecular Devices). Mouse sera that did not show reactivity to human IgG were pooled and further analyzed. Protein concentration of pooled sera was determined with commercial BCA assay to be 90 mg/ml in the WT and 92 mg/ml in the *Cmah*-KO.

### DMB-HPLC Analysis

The Sia content of mouse serum glycoconjugates was analyzed. Sias were released from glycoconjugates by acid hydrolysis using 0.1 M H_2_SO_4_ for 1 hours at 80°C. Free Sias were then derivatized with 1,2-diamino-4,5-methylenedioxybenzene (DMB) and cleared from proteins by Microcon-10 centrifugal filters then analyzed by fluorescence detection on reverse-phase HPLC (DMB-HPLC). Quantification of Sias was done by comparison with known quantities of DMB-derivatized Neu5Ac [38]. Mouse sera Sia content from WT and *Cmah*-KO was 2.5±0.2 mM and 2.1±0.5 mM, respectively. This is equivalent to 27±2 pmole Sia/µg protein in WT and 23±5 pmole Sia/µg protein in *Cmah*-KO (on average 91±2 µg/µl, 2.3±0.4 mM and 25±4 pmole Sia/µg protein for both strains).

### Mass Spectrometry Analysis

Analysis was done by the UCSD Glycotechnology Core Resource. *N*-Glycans were released from pooled mouse sera by PNGase-F [39] and *O*-glycans were released by alkaline reductive beta-elimination [40]. These were then permethylated and analyzed by MALDI-TOF and specific peaks confirmed by tandem mass-spectrometry (ms/ms) [41].

### ELISA

We tested binding of human serum and lectins to mouse serum glycoconjugates by ELISA. Costar 96-well were coated overnight at 4°C with saturating concentration (1 µg/well) of WT or *Cmah*-KO pooled mouse sera (lacking mouse-anti-human IgG) or alpha-Gal linked to polyacrylamide (250 ng/well; Galα1-3Galβ1-4GlcNAc-PAA) in coating buffer (50 mM sodium carbonate-bicarbonate buffer, pH 9.5). Wells were blocked for 2 hours at RT with PBS/OVA (PBS pH 7.3, 1% chicken ovalbumin (Sigma)). After removal of buffer, human sera (1∶100 diluted in PBS/OVA) or lectins (0.1 µg/ml Bio-SNA, 4 µg/ml Bio-MAL-1, 4 µg/ml Bio-MAL-2) were added to triplicate wells at 100 µl/well then incubated at RT for 2 hours. Wells were washed three times with PBST (PBS pH 7.3, 0.1% Tween-20), detection antibody was then added (100 µl/well, 1∶7,000 HRP-goat-anti-human IgG or 1∶10,000 HRP-streptavidin (0.1 µg/ml) diluted in PBS) and incubated for 1 hour at RT. After washing three times with PBST, wells were developed with 0.5 mg/ml *O*-phenylenediamine in citrate-PO_4_ buffer, pH 5.5, reaction was stopped with H_2_SO_4_ and absorbance was measured at a 490 nm wavelength on a SpectraMax 250 (Molecular Devices).

### Mild Periodate Treatment

To ascribe Sia specificity, mouse serum-coated ELISA plates were treated with mild periodate to selectively cleave the Sia side-chain [42]. Costar 96-well plates were coated overnight at 4°C with mouse sera as above. Next, wells were decanted and freshly prepared periodate solution (cold 2 mM sodium metaperiodate in PBS pH6.5, 200 µl/well) was added followed by gentle shaking for 20 min at 4°C in the dark. The reaction was stopped by addition of 50 µl of freshly prepared 100 mM sodium borohydride in PBS pH 6.5 (final concentration of 20 mM) followed by 10 min incubation at RT with gentle shaking (the borohydride inactivates the periodate). Concurrently, as a mock control, periodate and borohydride solutions were premixed at the same ratio (4∶1) and wells incubated (250 µl/well) side-by-side with the periodate-treated wells. To remove resulting borates, wells were washed three times (10 min each wash) with wash buffer (50 mM sodium acetate pH 5.5, 100 mM NaCl) then three times with PBS pH 7.3. Subsequently, wells were blocked and further processed as described (ELISA/EIA assays).

### Base Treatment

To remove 9-*O*-acetyl esters and render all Sias sensitive to mild periodate treatment, mouse serum-coated ELISA plates were base treated with 100 µl/well 0.1 M NaOH (final) at 37°C for 30 min, followed by neutralization with 100 µl/well 0.1 M HCl (final). Subsequently, plates were washed three times with PBS pH 7.3 then with PBS pH 6.5, followed by mild periodate treatment and development of ELISA with lectins as detailed.

### ELISA Inhibition Assay (EIA)

We tested human serum binding specifically to WT mouse serum Neu5Gc-glycoconjugates by EIA. Costar 96-well were coated overnight at 4°C with saturating concentration (1 µg/well) WT pooled mouse sera (lacking mouse-anti-human IgG) in coating buffer (50 mM sodium carbonate-bicarbonate buffer, pH 9.5). Wells were blocked for 2 hours at RT with PBS/OVA (PBS pH 7.3, 1% chicken ovalbumin). During the blocking, human serum was diluted 1∶100 in EIA buffer (PBS pH 7.3, 1% chicken ovalbumin and *Cmah*-KO pooled sera diluted at 1∶4000, thus containing ∼2.3 µg protein/100 µl and ∼57 pmole Sia/100 µl) and incubated on ice for 2 hours. Next, PBS/OVA was removed from wells and pre-treated human serum was added to triplicate wells at 100 µl/well then incubated at RT for 2 hours. Wells were washed three times with PBST (PBS pH 7.3, 0.1% Tween-20) then detection antibody was added (100 µl/well HRP-goat-anti-human IgG 1∶7,000 diluted in PBS) and incubated for 1 hour at RT. After washing three times with PBST, wells were developed with 0.5 mg/ml*O*-phenylenediamine in citrate-PO_4_ buffer, pH 5.5, reaction was stopped with H_2_SO_4_ and absorbance was measured at a 490 nm wavelength on a SpectraMax M3 (Molecular Devices). For Quantification of overall anti-Neu5Gc reactivity, each plate was also coated overnight with serial dilutions of purified human Igs (IgA and IgG; 10–0.3 ng/well). The Igs were coated side-by-side with WT mouse then all human serum samples and Ig standard were developed on the same plate for each Ig with HRP-goat-anti-human IgG or IgA.

### Human Sera Samples

Patients were enrolled at Rady Children’s Hospital, San Diego after obtaining parental informed consent and subject assent, as appropriate. The study protocol was reviewed and approved by the Institutional Review Board. All Kawasaki disease (KD) patients (n = 10) met the standard case definition for KD: had fever and at least 4 of the 5 standard clinical criteria (rash, conjunctival injection, cervical lymphadenopathy, changes in the oral mucosa, and changes in the extremities) or fever and 3 criteria plus coronary artery abnormalities (dilatation or aneurysm; [26]) documented by echocardiography. Only patients diagnosed within the first 10 days after fever onset were analyzed. Sequential samples were obtained from KD patients with normal course of disease (n = 5) or with coronary artery damage (n = 5) at three time points: acute phase, 2 weeks later (sub-acute) and at convalescent (1–3 months later). For controls we used age-similar healthy controls (HC) or febrile controls (FCs). FC were children evaluated in the emergency department who had fever for at least 3 days and diagnosed with Epstein-Barr virus (EBV, n = 3) or herpes simplex virus (HSP, n = 3). Whole blood was collected in red top tubes and the serum was separated from the clot within one hour of phlebotomy. Serum was stored at 4C for up to 72h and then frozen at −80°C until use.

### Sialoglycan Microarray Fabrication

Arrays were printed on Epoxide-derivatized Corning slides as described [21] with some modifications to print layout as described [37]. Epoxide-derivatized Corning slides were purchased from Thermo Fisher Scientific (Pittsburgh, PA) and the arrays were printed with SMT-S50, Classic silicon pins with 50×50 µm tips from Parallel Synthesis Technologies (Santa Clara, CA) using a UCSF DeRisi Linear Servo Motor Microarrayer, generating ∼70 µm diameter spots. Glycoconjugates were distributed into a 384-well source plates using 8 replicate wells per sample and 5 µl per well (Version 11P2). Each glycoconjugate was prepared at 100 µM in an optimized print buffer (300 mM phosphate buffer, pH 8.4). To monitor printing quality we used replicate-wells of human IgG (Jackson ImmunoResearch) at 150 µg/ml (in PBS) for each printing-pin. 8 pins were used, with each pin printing 6 replicate spots/well (by using the 384-well source plate 6 times), 24 spots/row with spacing of 180 mm. One complete array was printed with each pin generating 8 sub-array blocks on each slide (within approx 16 hours/∼100 slides). The humidity level in the arraying room was maintained at about 70% during printing. Printed slides were left on arrayer deck over-night, allowing humidity to drop to ambient levels. Next, the print order of the slides was recorded (using Fine Tip Black lab marker from VWR) and slides were packed, vacuum-sealed and stored in a desiccant chamber at RT until used. Slides were printed in one batch of ∼100 slides.

### Array Binding Assays

Each serum was tested in two duplicate sub-arrays on two different slides and data was averaged. Slides were incubated for 1 hour in a staining dish with 50°C pre-warmed blocking solution (0.05 M ethanolamine in 0.1 M Tris, pH 9.0) to block the remaining reactive groups on the slide surface, then washed twice with 50°C pre-warmed dH_2_O. Slides were centrifuged at 200×*g* for 3 min. Slides were then fitted with ProPlate™ Multi-Array slide module (Invitrogen) to divide into the sub-arrays then blocked with 200 µl/sub-array of blocking buffer (PBS/OVA, 1% w/v ovalbumin in PBS pH 7.3) for 1 hour at room temperature (RT) with gentle shaking. Next, the blocking solution was aspirated and 1∶100 diluted human serum samples were added to each slide (in PBS/OVA, 200 µl/sub-array) and allowed to incubate with gentle shaking for 2 hours at RT. Slides were washed three times with PBST (PBS containing 0.1% Tween) then with PBS for 10 min/wash with shaking. Bound antibodies were detected by incubating with 200 µl/sub-array of the Cy3-goat-anti-human IgG (H+L) at 1∶500 diluted in PBS (1.5 µg/ml) at RT for 1 hour. Slides were washed three times with PBST (PBS, 0.1% Tween) then with PBS 10 min/wash followed by removal from ProPlate™ Multi-Array slide module and immediately dipping slide in a staining dish with dH_2_O for 10 min with shaking, then centrifuged at 200×*g* for 3 min. Dry slides were vacuum-sealed and stored in dark until scanning the following day.

### Array Slide Processing

Processed slides were scanned and analyzed as described [21], [37] at 10 µm resolution with a Genepix 4000B microarray scanner (Molecular Devices Corporation, Union City, CA) using 350 gain. Image analysis was carried out with Genepix Pro 6.0 analysis software (Molecular Devices Corporation). Spots were defined as circular features with a variable radius as determined by the Genepix scanning software. Local background subtraction was performed.

#### Statistical analysis

Statistical analysis conducted with Prism 5 with the specific methods as indicated in the figure legends.

## Results and Discussion

### Principles for Simple Detection of Human Anti-Neu5Gc-Antibody Reactivity

**Figure 1:**
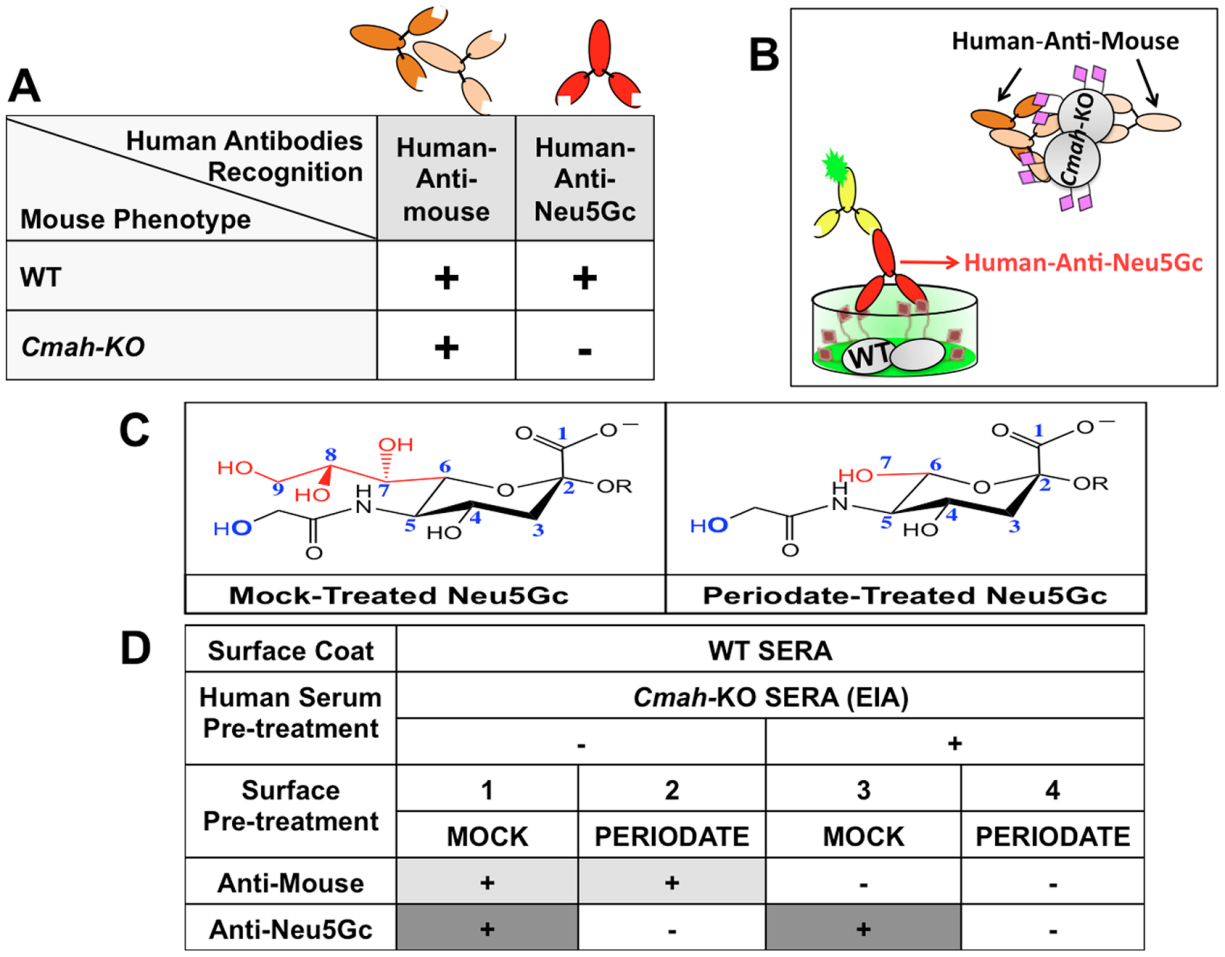
A simple method to detect overall human anti-Neu5Gc reactivity. **A.** Human antibodies can bind to mouse glycoproteins from WT (Neu5Gc-positive) or *Cmah*-KO (Neu5Gc-deficient) mice revealing anti-Neu5Gc and/or general anti-mouse reactivity. **B.** Illustration of the principles of EIA method that can separate specific anti-Neu5Gc reactivity from anti-mouse reactivity. Human antibodies are pre-incubated with *Cmah*-KO sera to absorb out anti-mouse reactivity, and binding to WT sera then reveals Neu5Gc-reactivity (grey circles = mouse glycoproteins; purple diamonds = Neu5Ac-containing glycoconjugates; red diamonds = Neu5Gc-containing glycoconjugates). **C.** Treatment of WT sialo-glycoconjugates with mild periodate cleaves the sialic acid side chain. **D.** Periodate pre-treatment of glycocojugates can demonstrate sialic acid specific binding but has no effect on anti-mouse reactivity.

To detect overall anti-Neu5Gc antibody reactivity in human samples we developed a simple method in which we exploit two mouse models that differ only by Neu5Gc-presence (wild-type; WT) or Neu5Gc-absence (*Cmah^−/−^* knockout; *Cmah*-KO). Humans have circulating antibodies recognizing various antigens, including anti-Neu5Gc antibodies and possibly human-anti-mouse antibodies. We hypothesized that when human antibodies reactivity is tested with WT mouse sample as target, both types of antibodies might be detected (Fig. 1A). However, when tested on *Cmah*-KO mouse sample as target, only human-anti-mouse reactivity would be detected with no anti-Neu5Gc reactivity, due to the lack of Neu5Gc-epitopes (Fig. 1A). This difference could allow use of *Cmah*-KO samples as an inhibitor for human-anti-mouse reactivity, thereby leaving only signal that is highly specific for Neu5Gc-epitopes and that could be revealed by appropriate tagged-detection system (Fig. 1B). To further confirm the signal is Sia-specific, mild periodate oxidation can be used to truncate the Sia side chain (Fig. 1C). This would specifically abolish Sia-dependent binding but not human-anti-mouse reactivity (Fig. 1D). To demonstrate this method, we chose to use mouse serum from both strains in an ELISA inhibition assay based format (EIA) to detect human serum anti-Neu5Gc antibodies on coated WT mouse serum, using *Cmah*-KO mouse serum to inhibit non-Neu5Gc reactivity (Fig. 1B–D).

### Characterizing Serum from Wild-Type and *Cmah*-KO Mice

**Figure 2:**
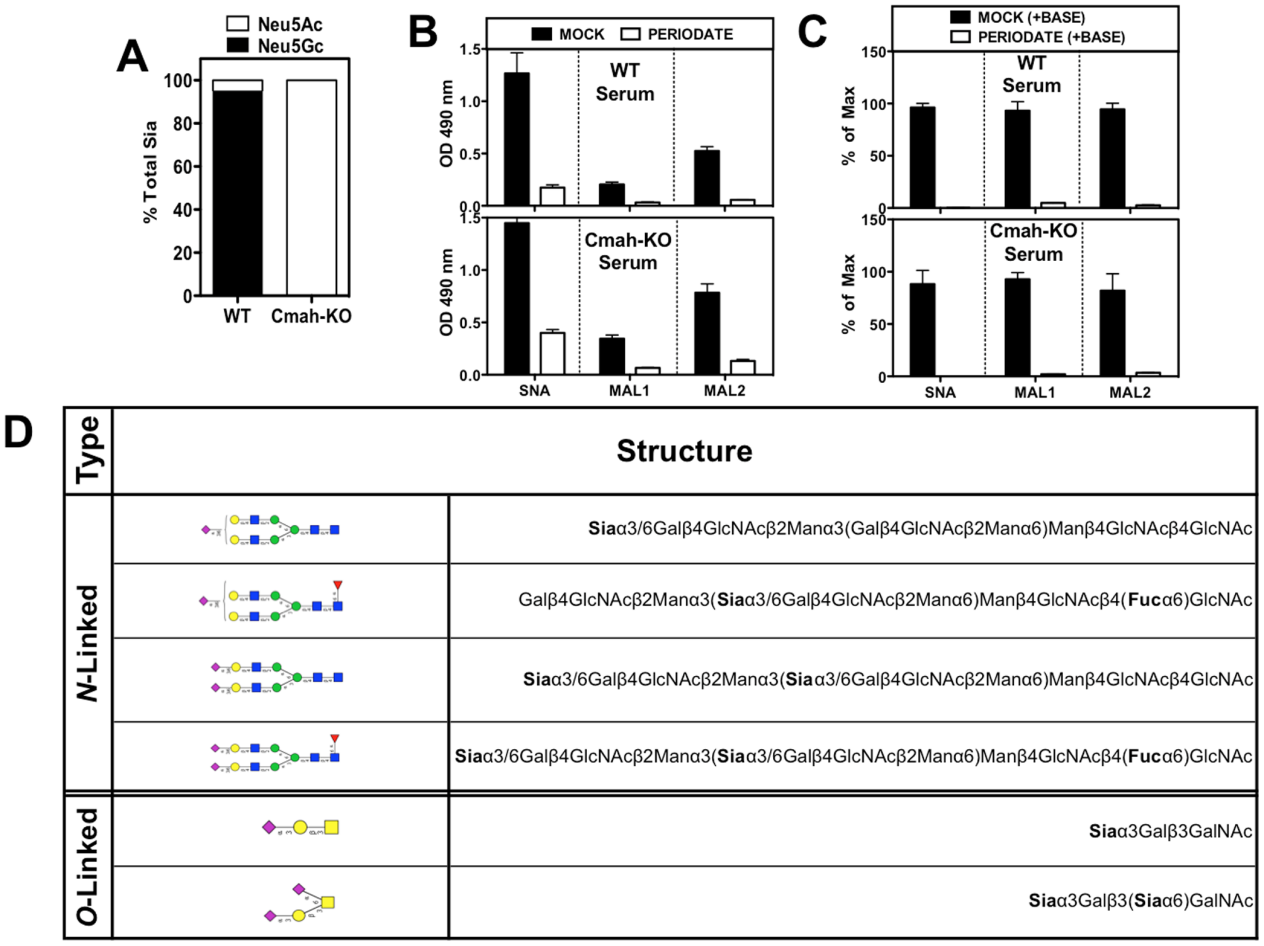
Sialic acid characteristics of sera from WT and *Cmah*-KO Mice. A. DMB-HPLC analysis of mouse sera confirms that WT is Neu5Gc-positive while *Cmah*-KO is Neu5Gc-deficient. **B.** Lectin binding on mouse sera demonstrates both Siaα2-6 (SNA) and Siaα2-3 (MAL1/2) glycoconjugates in both WT and *Cmah*-KO. After periodate treatment the signal is reduced demonstrating Sia specificity. The periodate-resistant signal suggest 9-*O*-acetylated Sias. **C.** Pre-treatment of coated-wells with base removes periodate resistant signals (white bars in B compared to C), confirming that some of the Sia are modified with 9-*O*-acetylation. **D.** Mass-spectrometry analysis of *N*-linked and *O*-linked glycans of WT and *Cmah*-KO sera reveals the most common sialoglycan structures. Each figure is representative of at least two independent experiments. Bars are mean ± s.d. of triplicates (A–C).

To allow use of mouse serum as targets for human antibodies binding, we initially screened individual mouse sera for reactivity against human antibodies (data not shown), and then used only those that do not show mouse-anti-human reactivity (to avoid false positives). Such mouse sera were collected and pooled from either WT or *Cmah*-KO mice. The sera from both strains are expected to have similar protein profiles yet different glycosylation patterns, mainly the presence or absence of Neu5Gc, due to deactivation of the *CMAH* enzyme in the *Cmah*-KO. Analysis of the sialic acid (Sia) content by DMB-HPLC confirmed that *Cmah*-KO sera contain only Neu5Ac, while the WT sera contain mostly Neu5Gc (∼90±9%; Fig. 2A).

Sia carry a characteristic acidic carboxylate group and have a 9-carbon backbone. They normally cap glycoconjugates and are linked to underlying sugars by various linkages (Siaα2−3 to galactose, Siaα2−6 to galactose or *N*-acetylgalactosamine or Siaα2−8 to another Sia) [2]. To evaluate the Neu5Gc-epitopes targets in WT mouse sera, we first analyzed the Sia-linkage on serum sialoglycoproteins by ELISA using the Sia-specific lectins SNA (specific for Siaα2-6), MAL-1 and MAL-2 (both specific for Siaα2-3) [37], [43], [44]. This analysis revealed that both WT and *Cmah*-KO sera contain both Siaα2-3/6-linked glycoconjugates with the Siaα2-6-linked being more abundant (Fig. 2B). To demonstrate Sia-specificity, we used mild periodate oxidation followed by reduction with borohydride that specifically generates a truncated-Sia with a terminal hydroxyl at carbon 7. The resulting modified Sia has two less carbons (carbons 8 and 9), but maintains the negative charge (Fig. 1C) [37], [45]. Lectin binding was reduced after periodate treatment of the coated sialoglycoconjugates, demonstrating Sia-specificity (Fig. 2B). The residual low lectins binding observed after periodate treatment suggests the presence of *O*-acetylated Sia (Fig. 2B). Sia can be modified by *O*-acetyl esters at positions C4, C7, C8, and/or C9 (Fig. 1C). C9 esters can serve as protecting groups rendering *O*-acetylated-Sia insensitive to periodate oxidation [46], [47]. However, periodate sensitivity can be restored if the *O*-acetyl esters are removed by mild base treatment [46], [47]. Indeed, when the coated sialoglycoconjugates were first treated with base then followed by periodate treatment, residual lectin binding was completely abolished (Fig. 2C), thereby verifying the presence of *O*-acetylated-Sia in mouse sera. Finally, mass-spectrometry analysis of *N*-linked and *O*-linked glycans of WT and *Cmah*-KO sera revealed the most common sialoglycan structures. This included mono/di-sialylated bi-antennary *N*-glycans (fucosylated or non-fucosylated) and linear or branched sialylated *O*-glycans (Fig. 2D). In WT sera, Neu5Gc:Neu5Ac ratio is 9∶1 for *N*-glycans and 5∶1 for *O*-glycans. In general, the *N*-glycans are more abundant than the *O*-glycans (∼×30 higher in the WT sera).

### Detection Of Specific Anti-Neu5Gc Antibody Reactivity in Human Serum: Proof of Principle

**Figure 3:**
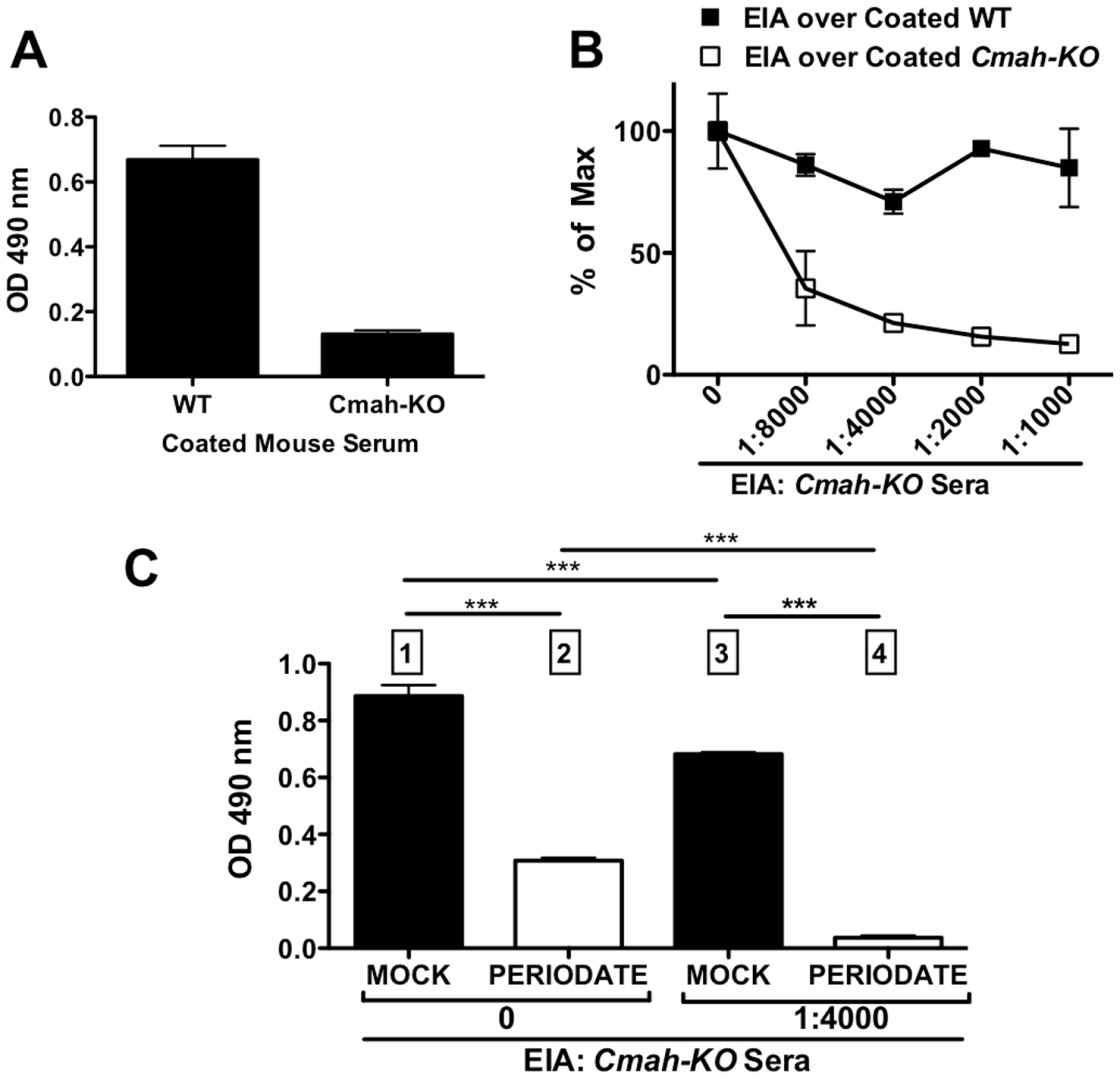
Specific detection of anti-Neu5Gc IgG in normal human serum. **A.** Plates were coated with WT or *Cmah*-KO mouse sera and Anti-Neu5Gc IgG of human serum S34 [16] tested by ELISA. **B.** Plates were coated with WT or *Cmah*-KO mouse sera, then human serum S34 IgG binding tested by ELISA (0) or by EIA after pre-incubation of S34 with *Cmah*-KO mouse sera (as depicted in Fig. 1B). EIA specifically inhibited anti-*Cmah*-KO (anti-mouse) reactivity, resulting in Neu5Gc-specific reactivity on WT coated plates. For S34, the optimal *Cmah*-KO inhibitor is at 1∶4000 dilution. **C.** Sialic acid specific binding demonstrated by mock versus mild periodate pre-treatment of coated wells. Human serum S34 (1∶100) tested over WT mouse serum without inhibitor (0) or by EIA method with diluted *Cmah*-KO mouse sera. In the presence of *Cmah*-KO inhibitor, S34 binding to WT was abolished compared to mock treated wells hence specificity is towards sialylated-(Neu5Gc)-glycans. Numbers 1–4 refer to the specific steps predicted by the model, as described in Fig. 1D. Each figure is representative of at least two independent experiments. Bars are mean ± s.d. of triplicates (A–C).

To demonstrate this method we tested a previously characterized normal human serum (S34) that has high IgG reactivity with multiple Neu5Gc-epitopes [16], [21]. In ELISA, S34 showed strong IgG binding to the Neu5Gc-positive WT mouse sera and lower binding to the Neu5Gc-deficient *Cmah*-KO mouse sera (∼20% of WT; Fig. 3A). This suggested strong reactivity to Neu5Gc-epitopes with low binding to other mouse epitopes. We then used ELISA inhibition assay (EIA), in which S34 was pre-incubated with serially diluted *Cmah*-KO mouse sera to remove human-anti-mouse reactivity, and it was then allowed to bind mouse sera coated targets. As the concentration of inhibitor increased, S34 showed dramatically reduced binding to *Cmah*-KO sera coated targets (Fig. 3B). In contrast, on WT sera coated targets, there was ∼20% maximal decrease in S34 binding with an optimal range of diluted *Cmah*-KO inhibitor 1∶4000 for S34; Fig. 3B). To further prove Sia-specificity, mild periodate treatment was used in the presence or absence of *Cmah*-KO inhibitor (Fig. 3C). This clearly demonstrated the loss of anti-mouse reactivity only when inhibitor was present and the abolishment of binding signal after mild periodate treatment, thus indicating specific anti-Neu5Gc reactivity (Fig. 3C). These findings matched our hypothesis as outlined in Fig. 1 and further confirmed for IgG and IgA using other normal human sera (data not shown). Of note, S34 has strong anti-Neu5Gc IgG reactivity [16] and low reactivity to mouse sera antigens. Other sera may have different levels of reactivities to these antigens (higher/lower for both antigens) thus the concentration of the inhibitor may need to be optimized to match such different profiles.

### Anti-Neu5Gc Antibodies Reactivity in Kawasaki Disease

To validate the simple detection of such antibodies in a disease context, we tested serum from patients with KD that is the leading cause of acquired heart disease in children [23], [24]. We hypothesized that anti-Neu5Gc antibodies may be detected in KD patients for several reasons. First, these antibodies appear in infants coinciding with exposure to Neu5Gc-containing foods (baby formula and red meat products) [18]. Second, anti-Neu5Gc antibodies have been shown to contribute to human cardiovascular diseases [22]. Lastly, it has been suggested that heterophile antibodies to H-D antigen, that presumably contained Neu5Gc, are elevated in KD patients [33]–[35].

**Figure 4:**
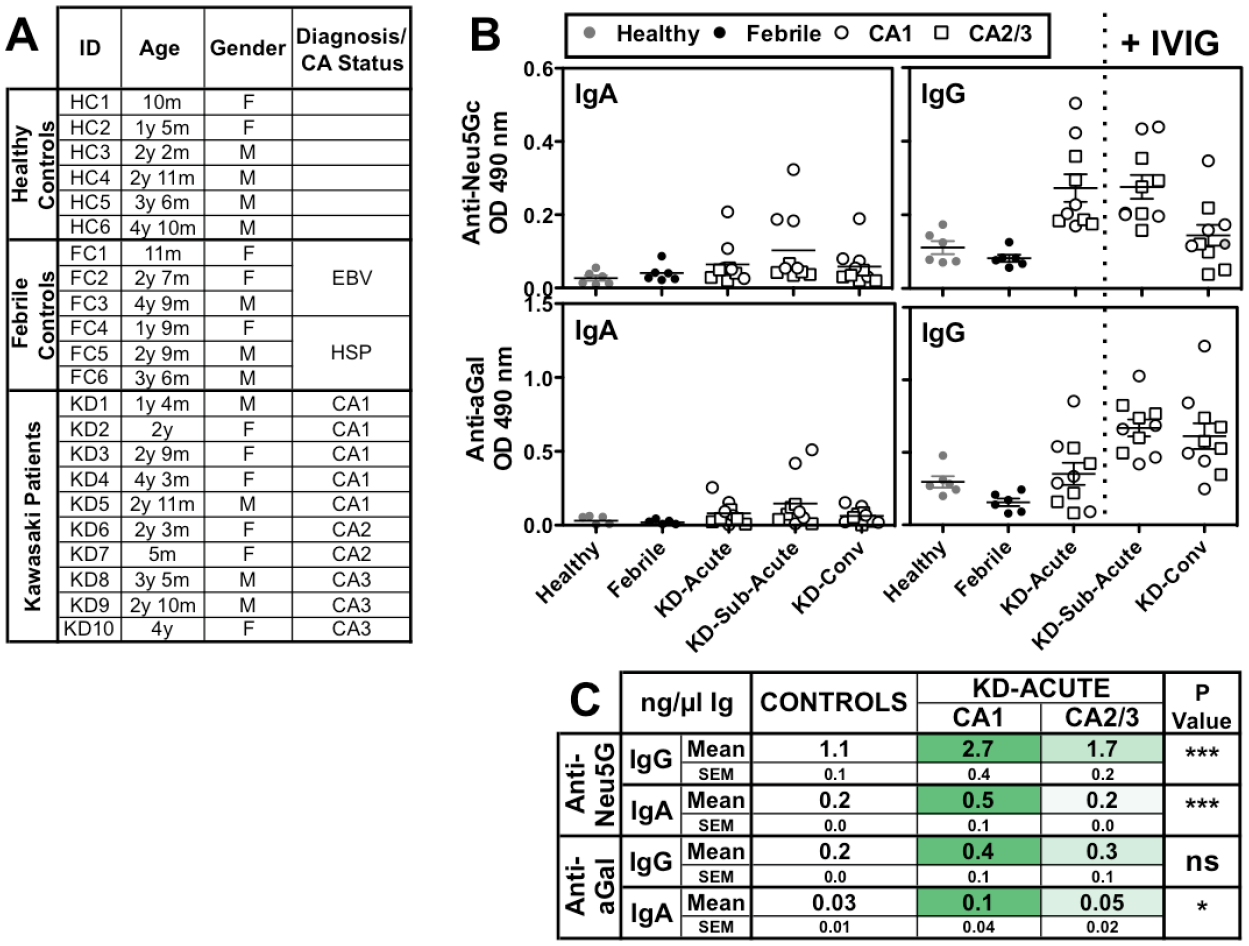
Characterization of anti-Neu5Gc IgA and IgG in Kawasaki disease patients’ sera. A. Details of the cohort of sera used for analysis of antibodies. Coronary arteries (CA) status represents normal (CA1) or damaged with aneurysm (CA2) or dilatation (CA3). **B.** Analysis of IgA and IgG antibodies against Neu5Gc-epitopes (by EIA over WT sera) and alpha-Gal-epitope (by ELISA) in controls versus KD patients’ sera at acute (at diagnosis prior to treatment), sub-acute (at 2 weeks post-IVIG) and convalescent phases (1–3 months post IVIG) of disease. KD sera revealed increased antibodies responses in the sub-acute compared to the acute phase (repeated measures One-Way ANOVE with Tukey’s multiple comparison test: anti-Neu5Gc IgA P = 0.0213, IgG P = 0.0003; anti-alpha-Gal IgA ns, IgG P<0.0001). Representative of two independent experiments (data for each individual is provided in Fig. S1). **C.** Quantitative analysis of anti-Neu5Gc (EIA) and anti-alpha-Gal (ELISA) IgA and IgG responses in sera samples conducted against the respective immunoglobulin (Ig) standard curves. All sera samples were tested in duplicates on a single plate for each Ig. KD sera with CA1, but not with CA2/3, show significant increased antibodies levels compared to controls as summarized (One-Way ANOVA with Tukey’s multiple comparison test; anti-Neu5Gc IgA P = 0.0194, IgG P = 0.0045; anti-alpha-Gal IgA P = 0.0124, IgG P = 0.1479). Data from two independent experiments.

A total of 42 sera samples were obtained from 10 KD patients and 12 controls (as detailed in Fig. 4A). Sera were serially collected from KD patients (age range 5m –4y3m) at three stages of the disease: before treatment with IVIG during the acute phase and after treatment with IVIG at 2 weeks (sub-acute phase) and 1–3 months (convalescence phase). In addition, KD patients were selected to represent various degrees of coronary artery (CA) status during disease progression. Five of the KD patients had normal CA (CA1), two had aneurysms (CA2) and three had dilated arteries (CA3). The controls were comprised of age-matched sera from 6 healthy children (age range 10m –4y10m), and 6 febrile patients (age range 11m –4y9m) diagnosed with either Epstein-Barr virus (EBV) or herpes simplex virus (HSP) (Fig. 4A).

Serum samples were qualitatively analyzed by EIA to detect overall anti-Neu5Gc IgA and IgG antibodies at three stages of the disease (acute, sub-acute, convalescence) compared to the controls (Fig. 4B). This revealed an elevated response in KD patients compared to all control sera in the acute phase. Anti-Neu5Gc IgA showed further increase in the sub-acute phase that began to resolve at convalescence. A similar trend was also seen for anti-Neu5Gc IgG, although in this case the elevated response may have been partly attributed to the administered therapeutic human IgG (IVIG) that contains anti-Neu5Gc IgGs [17] (Fig. 4B). We also tested by ELISA the common antibodies to the related carbohydrate xeno-antigen, alpha-Gal [19], [48]. Anti-Neu5Gc and anti-alpha-Gal antibodies are both generated in humans during infancy [18]. However, in contrast to the variable nature of Neu5Gc-epitopes, the alpha-Gal epitope is a single structure (Galα1-3Galβ1-4GlcNAc-R) [48] [19]. In general, anti-alpha-Gal IgA and IgG showed similar antibody response profiles to anti-Neu5Gc antibodies (Fig. 4B). Taken together, these data suggest that the elevated anti-Neu5Gc antibody responses are not unique, but rather fit with previous observations of an overall polyclonal B-cell response [30]–[32]. However, we noticed that those elevated responses were largely observed in KD patients with normal coronary arteries (CA1) rather than with damaged ones (CA2/3) (EIA assay conducted while blinded to CA status, that was revealed only after data analysis). Subsequent quantitative analysis of these antibodies in the acute phase demonstrated that compared to control sera there is significant elevated anti-Neu5Gc IgA and IgG only in patients with CA1 but not with CA2/3 (Fig. 4C). In line with IgG and IgA isotype relative abundance in sera [49], [50], anti-Neu5Gc IgG levels were greater than those of anti-Neu5Gc IgA. Although anti-alpha-Gal antibodies showed a similar profile, their levels were ∼5 fold lower compared to anti-Neu5Gc antibodies (Fig. 4C). The alpha-Gal epitope is not expressed in humans (or other old-world primates) and cannot be taken up through the diet, but is rather immunogenic on gut bacteria [19]. In contrast, Neu5Gc is an immunogenic dietary carbohydrate that metabolically incorporates into human cells with its subsequent presentation on multiple glycoconjugates on the cell surface and secreted glycoconjugates [9]–[11]. In mouse models, the role of anti-Neu5Gc antibodies in cancer may be determined by their levels, with low levels stimulating disease [20] but high levels inhibiting tumor growth [21]. In addition, patients with metastatic carcinomas may have lower levels of anti-Neu5Gc antibodies, possibly due to their absorption by tumor cells on elevated tumor burden [21]. Therefore, variable levels of anti-Neu5Gc antibodies in KD patients may have a biological significance.

**Figure 5:**
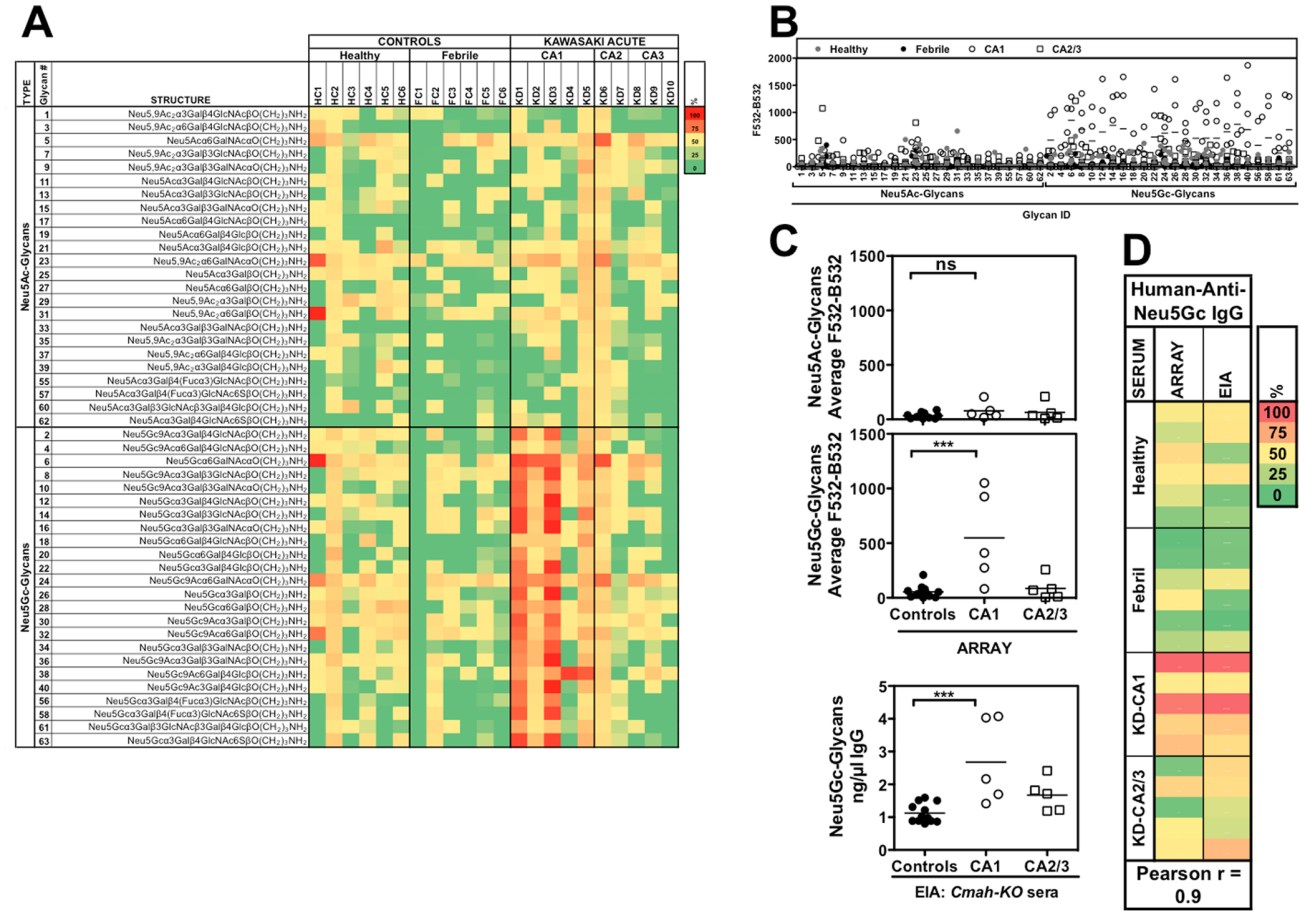
Anti-Neu5Gc IgG profile analysis by sialoglycan microarray versus EIA in acute phase KD. A–B. Sialoglycan microarray analysis of sera from controls (healthy or febrile) or from acute phase KD patients with either normal (CA1) or damaged (CA2/3) coronary arteries. Data is presented as a heatmap of percentile reactivity (A) or summarized as fluorescent signal per glycan (data for each individual is provided in Fig. SB). (B) and shows greater reactivity to Neu5Gc-glycans in KD sera with CA1 compared to CA2/3 or the controls. Representative of two independent experiments. **C.** Comparison of antibodies reactivity, as tested by microarray or by EIA, between controls and acute KD sera with normal or damaged CA. Microarray data for all Neu5Ac/Neu5Gc-glycans was averaged per donor sera and compared between groups. EIA data in ng/µl IgG was averaged from two independent experiments. Statistical analysis revealed a significant difference only for Neu5Gc-glycans between febrile controls and KD patients with CA1 (One-Way ANOVA with Tukey’s multiple comparison test; Array P = 0.0007; EIA P = 0.0014). Data is representative of at least two experiments. **D.** Correlation analysis of anti-Neu5Gc IgG reactivity obtained by EIA (ng/µl IgG) versus sialoglycan microarray (average F532-B532 of all Neu5Gc-glycans per serum sample).

To independently validate these findings, we conducted a more detailed analysis of anti-Neu5Gc IgG in controls and acute-KD sera samples on a sialoglycan microarray. This array is printed with various natural Neu5Gc-glycans side-by-side with their matched Neu5Ac-glycans that differ by only a single oxygen atom [21], [37]. Consistent with the antibody reactivity by EIA (Fig. 4C), binding profiles on the array revealed distinctively stronger broad reactivity to multiple Neu5Gc-glycan epitopes in KD sera with CA1 compared to CA2/3 or the controls, while no significant differences observed for the corresponding control Neu5Ac-glycans (Fig. 5A–B). This broad binding profile suggests that while these antibodies are highly specific for Neu5Gc-containing epitopes, many of them are likely cross-reacting with several distinctive Neu5Gc-epitopes. To qualitatively compare between the detection of antibodies by sialoglycan microarray versus the newly developed EIA, the antibody reactivity on Neu5Ac/Neu5Gc-glycans by microarray was averaged per donor sera and compared between the sera groups. Statistical analysis revealed a significant difference only for Neu5Gc-glycans between controls and KD patients with CA1 (Fig. 5C). This profile exactly matched the profile of anti-Neu5Gc IgG obtained by the EIA method (Fig. 5C). Moreover, direct comparison of these anti-Neu5Gc IgG values obtained by the two methods showed strong correlation (Fig. 5D). The differences in antibody response among the 3 KD groups based on coronary artery status may suggest that immunologic differences are linked to disease outcome. Altogether, these findings strongly support the EIA as a valid and rapid method for detection of overall anti-Neu5Gc reactivity in human sera samples.

### Conclusions

Detection of overall anti-Neu5Gc reactivity needs to be studied as a potential method to detect risk, progression or therapeutic responses in chronic inflammation-related diseases such as cancer and cardiovascular diseases. Here we describe a rapid and efficient method to detect overall anti-Neu5Gc responses in human sera. We have developed this method by using WT and *Cmah*-KO mouse sera (respectively Neu5Gc-positive or -negative), which contain multiple Sia-epitopes with either native- or modified-Sia (*O*-acetylated) that are variously linked to underlying glycans. Similarly, WT and *Cmah*-KO matched sera from other organisms (e.g., porcine) can be used. In fact, any matched tissue from WT and *Cmah*-KO can theoretically be used, because the presence and absence of Neu5Gc is likely respectively maintained. Different matched-tissues/organisms may represent additional/other Neu5Gc-epitopes not necessarily present in sera, and should be studied in the future. Here, we also used the *Cmah*-KO sera as ‘in-solution’ inhibitor to block non-Sia binding and facilitate rapid detection. However, human-anti-mouse reactivity might also be removed from candidate human sera prior to the detection assay, for example by attaching the *Cmah*-KO sera inhibitor to magnetic beads (or other solid surface) for pre-absorption. In addition, other detection methods can be used instead of ELISA; for example dot blot that would result in a deposition of insoluble color on a membrane leaving a colored dot where anti-Neu5Gc reactivity is detected (much like a ‘pregnancy-test’). This method might also be applicable to specifically detect human anti-Neu5Gc reactivity in various human samples other than serum, such as plasma, synovial fluid, tissue extracts etc. This rapid and robust assay can now be used for detection of overall anti-Neu5Gc reactivity as a potential screening method to monitor risk, progression or therapeutic responses in various inflammation-mediated conditions.

## Supporting Information

Figure S1 **Full data.**
(XLSX)
